# Translations

**Published:** 1882-04

**Authors:** Seth N. Jordan

**Affiliations:** Columbus, Ga.


### Translations.

By SETH N. JORDAN, M.D., Columbus, Ga.

*From Foreign Journals for the Atlanta Medical Register.*

*A Contribution to the Physical Examination of the Blood-Vessels. N. Friedrich Arch. f. Klin. Med. XXIX, p.* 256.—I. The humming-top murmur and its diagnostic importance.—F. differentiates between weak and strong humming-top murmurs in the internal jugular. The strong ones are easily distinguished by palpation, and are characteristic of anæmic and chlorotic conditions. Weak murmurs of this nature are sometimes heard in healthy subsubjects. Humming-top murmurs, or as they are known by French authors, bruit de diable, can be heard in the vena cava superior, being in the first instance transmitted from the jugular vein, or can arise from compression of tumors or lung, or can be the result of chlorosis or anæmia. Venous murmurs in the brachial and axillary veins are rare, and occur only in hydræmic conditions. This same applies to murmurs in the crural vein. These last can be palpated.

II—Regurgitating Murmur and Expiratory Sounds in the Crural Vein.—During vigorous expiratory movements one can feel and hear a short stroke over the crural vein, which corresponds to the unfolding crural venous valves.

III.—Sounds and Murmurs in the Arteries.—F. inclines to the opinion that the first sounds over aorta and pulmonalis do not arise therein, but are transmitted from their respective ventricles. By pressure and displacement of an artery, as well as by the stroke of the artery against the stethoscope, a murmur may be produced. Over the abdominal aorta, transmitted and antochthonous sounds can be heard. The first coincides with the sounds of the heart. Spontaneous sounds can mingle with transmitted murmurs, thereby forming double and three-fold arterial sounds. By means of sufficient pressure a murmur is produced, and if at the same time the vena cava inferior be compressed, a venous-arterial or mixed epigastric double murmur arises. In the deeper sections of the abdominal aorta, such a double murmur does not occur, since both vessels cannot be compressed at once, and therefore here all double murmurs are arterial in nature.

---

*On the Treatment of Tumors by Electrolysis. B. Neftel. Virchow's Archives. LXXXVI.* I. For benign tumors the author employs the following methods: 1. During local anæsthesia the cathode needle is inserted into the tumor, while the flat anode is held stabile at a short distance. Sitting to last 5–10 minutes; repetition in 3–5 days. Later, after three or four such sittings, the treatment must be carried on with percutaneous application of both poles cathode and anode being flat. This percutaneous treatment can be entirely omitted in cases where sittings with insertion of needle have been carried from 4–10 times. The needle should each consecutive time pass in by means of the opening made at the first sitting, and each time it should be given a different direction. If it be desired to avoid all discharge through the opening made by the needle (in retro-uterine myomata for example) then the cathode should be introduced at intervals of 10–20 days through the vagina into the tumor, and the flat anode held on the sacrum; 30 elements are to be used, and each time the needle must be inserted in a new place. Neuromata are also treated thus successfully, many cases of which N. reports in the article by way of illustration. Electrolysis, according to Neftel, is antiseptic in its behavior: antisepsis produced by the ozone and hydrogen arising from the anode, hydrogen in an active form from the cathode, and free acids from the anode.

Electrolysis is accessible to all parts of the body, and can be employed in many instances in which other antiseptica could not be used. As is stated, the treatment of malignant and benign tumors by electrolysis is different in principle. In the treatment of malignant tumors there exists no method comparable to electrolysis.

---

*Carcinoma of the Vulva. O. Kustner, Centralblatt f. d. Med. Wissenschaften*, 1882, *No.* 6.—K. communicates five cases of carcinoma of the vulva, all of which were kept under observation after operation. Only one cure followed operation; since the operation three years have passed. In the other four cases a return of the tumor occurred soon after the operation, not only in the old scar, but in the inguinal glands, which were afterwards removed. Of these, on whom the operation had been performed for the second time, one died in one month, one in eight months, and the third one year after the second operation.

From the whole we conclude, that cancer of the exterior female genitals takes on very early the utmost pernicious character. The operation, therefore, is to be made as early and as thoroughly as possible. In case any of the inguinal glands are swollen all are to be removed—tne deep as well as the superficially located.

---

*Some Causes of Transitory Albuminuria. J. Fischl. Arch. f. Klin. Med., XXIX, p.* 217.—In several instances of diseases of the intestinal canal accompanied by violent paroxysms of pain, I was able to find albumen in the urine. It is requisite that the pain reach a certain intensity and be of sufficient duration. The albuminuria disappeared after a short period, rarely continuing for three weeks. In most of the cases epithelial cells and hyaline cylinders were present in small quantity. The greatest quantity of albumen was found in the urine voided immediately after the paroxysm of pain. Authors theory: pain in a reflex way brings about an arterial anæmia and venous hyperæmia of the blood-vessels of the kidneys—organic changes are to be excluded.

Fischl observed, also, albuminuria in one case of hemorrhage, and in a case of intestinal catarrh.

---

*Injection of a Solution of Salt Into the Artery. Bischoff, Centralblatt*, 1881, *No.* 23.—Induced by the favorable results of Schwarz on animals, B. infused into a patient greatly exhausted by post partum hemorrhage a solution of salt.

After ligating the central radial artery, 1250 grm. of a 0.6 per cent. solution of salt was allowed to flow in during the period of an hour. Patient recovered rapidly. Any unpleasant symptoms, as so often occur during transfusion of blood, did not take place. For future cases B. would prefer the hydrate of natrium.

---

*The Action of Quinia and Salicylic Acid on the Ear. Kirchner. Wochenschrift, Berlin*, 1881, *No.* 49.—Experiments on cats, dogs, mice, and rabbits, as to the change produced by quinia and salicylic acid on the organ of hearing, showed that these substances cause a hyperæmic condition of the labyrinth as well as the tympanum. Regarding the cause of these pathological changes, Kirchner advocates the opinion of Weber, Liel, and Roosa, that vaso-motor influences are the seat of these changes, and it must be accepted that a paralysis of the blood-vessels with stasis and exudation in the different tissues of the ear takes place.

---

*Galvanization of Regions Painful on Pressure. M. Meyer. Wochenschrift, Berlin*, 1881. *No.* 31.—Meyer has already repeatedly called attention to the diagnostic importance of these "points de douleur." Such places are generally located on or near the spinal column, and oftenest corresponding to the transverse process. Sometimes such painful points may be situate in the nerve plexus or at its entrance into the muscle. Many cases of violent neuroses Meyer has succeeded in relieving greatly by placing the anode on the point of pain, and cathode on the sternum, using at first from 4 to 8 elements.

---

*The Etiology of Tetanus and Trismus. G. Burmeister. Dissertation. Berlin,* 1881.—In four-fifths of the reports of autopsies at the Berlin Maternity, B. found arteritis umbilicalis the most prominent pathological change. All other changes the author attributed to the tetanus itself, to the secondary appearance of the arteritis, or, as usual, to the bodies of the new-born. The pleuritis, so often observed, is either a continuation of the inflammation in the umbilical vessels, or produced by pyæmic processes.

---

*Perforation of the Duodenum by Ascarides. Arch. f. Klin. Med. XXIX, p.* 601. *E. Marcus.*—A girl 13 years of age grew suddenly ill with symptoms of perforation-peritonitis. She died on the seventh day. At the autopsy three ascarides were found in the peritonitic exudation. In addition, there was found in the descending portion of the duodenum a slit 6 mm. long and 2 mm. wide, near which no ulceration could be found, so that its origin was declared to be "ascaridophagous."

